# *Desulfovibrio vulgaris* exacerbates sepsis by inducing inflammation and oxidative stress in multiple organs

**DOI:** 10.3389/fmicb.2025.1574998

**Published:** 2025-04-30

**Authors:** Rong Wu, Zhichao Yu, Peiheng Guo, Xiaopeng Xiang, Yunong Zeng, Shanshan Fu, Mei Yang, Xintao Huang, Ze Wang, Ali Chen, Yuewei Ge, Xiaoshan Zhao, Wei Xiao

**Affiliations:** ^1^School of Traditional Chinese Medicine, Southern Medical University, Guangzhou, China; ^2^Hong Kong University of Science and Technology, Guangzhou, China; ^3^School of Chinese Materia Medica, Guangdong Pharmaceutical University, Guangzhou, China; ^4^Department of Breast Cancer, Cancer Center, Guangdong Provincial People's Hospital, Southern Medical University, Guangzhou, China; ^5^Center for Drug Research and Development, Guangdong Provincial Key Laboratory for Research and Evaluation of Pharmaceutical Preparations, Guangdong Pharmaceutical University, Guangzhou, China; ^6^Key Laboratory of Glucolipid Metabolic Disorder, Ministry of Education, Guangdong Pharmaceutical University, Guangzhou, China

**Keywords:** sepsis, macrophage, *Desulfovibrio vulgaris*, inflammation, oxidative stress

## Abstract

**Introduction:**

Sepsis is a life-threatening condition that often leads to organ dysfunction and systemic inflammation, with gut microbiota dysbiosis playing a crucial role in its pathogenesis. The role of *Desulfovibrio vulgaris* (*D. vulgaris*), a potentially pathogenic bacterium, in sepsis remains unclear.

**Methods:**

We first assessed the abundance of *D. vulgaris* in the feces of septic mice and patients using qPCR. Mice were then orally gavaged with *D. vulgaris* (2 × 10^8^ CFU/mouse/day) for 7 consecutive days followed by cecal ligation and puncture (CLP) surgery. We monitored survival, assessed organ damage, and measured inflammation. Peritoneal macrophages were isolated to analyze the phosphorylation of key MAPK and NF-κB signaling pathways. Finally, oxidative stress levels in the liver, lungs, and kidneys were evaluated, measuring markers such as GSH, CAT, and SOD.

**Results:**

The abundance of *D. vulgaris* was significantly increased in the feces of both septic mice and patients. Supplementation with *D. vulgaris* exacerbated sepsis in mice, resulting in lower survival rates, more severe organ damage, and heightened inflammation. Phosphorylation of MAPK and NF-κB pathways in peritoneal macrophages was significantly enhanced. Additionally, *D. vulgaris* amplified oxidative stress across multiple organs, as indicated by increased ROS levels and decreased antioxidant enzyme activity.

**Conclusion:**

Our findings suggest that *D. vulgaris* exacerbates the progression of sepsis by enhancing inflammation, activating key immune signaling pathways, and increasing oxidative stress. These processes contribute to organ dysfunction and increased mortality, highlighting the potential pathogenic role of *D. vulgaris* in sepsis.

## Introduction

Sepsis and septic shock are major global healthcare challenges, with mortality rates exceeding 25%, resulting in substantial morbidity and healthcare costs (Fleischmann et al., 2016). Timely intervention is essential to improve survival outcomes in sepsis patients, as delayed treatment increases mortality by 4% per hour (Han X. et al., 2021). Mortality rates among critically ill sepsis patients vary based on factors such as sepsis severity, patient demographics, and healthcare settings. Recent studies have reported intensive care unit mortality rates for sepsis patients ranging from approximately 26–41.9% (Sakr et al., 2018; Fleischmann-Struzek et al., 2020).

Sepsis, as a systemic inflammatory response syndrome, involves complex immune, metabolic, and inflammatory regulatory networks (van der Poll et al., 2017; Zhang and Ning, 2021; Chen et al., 2022). The onset of sepsis is intricately linked to multiple factors (Giamarellos-Bourboulis et al., 2024), with gut microbiota dysbiosis emerging as a critical contributor (Haak and Wiersinga, 2017; Sun et al., 2023). In sepsis, gut microbiota undergoes profound disruption, compromising the intestinal barrier and triggering aberrant immune responses (Lankelma et al., 2017; Piccioni et al., 2024). Gut microbiota dysbiosis influences systemic immune activation through the “gut-immune axis” contributing to the intensification of inflammation, organ dysfunction, and poor clinical outcomes in sepsis patients. This dysbiosis may drive sepsis progression through mechanisms such as the release of pro-inflammatory cytokines, increased oxidative stress, and immune cell dysfunction (Liu W. et al., 2020; Fang et al., 2022; Chancharoenthana et al., 2023; Chen et al., 2023; Kunst et al., 2023). As a result, understanding the role of gut microbiota in sepsis has become a prominent area of investigation in recent years.

Among the gut microbiota, *Desulfovibrio* species are anaerobic, Gram-negative, and sulfate-reducing bacteria that play an important role in host health by influencing metabolism and immune responses (Figliuolo et al., 2017; Lu et al., 2023). *Desulfovibrio vulgaris* (*D. vulgaris*), a prominent species within this group, produces hydrogen sulfide (H₂S) through sulfate reduction (Ueki and Lovley, 2022). While low concentrations of H₂S serve as signaling molecules, higher concentrations can be toxic, damaging intestinal epithelial cells, compromising the gut barrier, and amplifying systemic inflammation (Jin et al., 2024; Munteanu et al., 2024a). These properties suggest a potential role for *D. vulgaris* in exacerbating conditions like sepsis, where immune responses and intestinal barrier dysfunction are critical factors.

*Desulfovibrio vulgaris* has garnered significant attention due to its potential pathogenic role in various diseases. Its overgrowth has been linked to the development of ulcerative colitis, inflammatory bowel disease, Parkinson’s disease, and colorectal cancer (Murros et al., 2021; An et al., 2023; Huang et al., 2024; Xie et al., 2024; Dong et al., 2025). *D. vulgaris* abundance is increased in patients with ulcerative colitis and correlates with the severity of the disease (An et al., 2023; Xie et al., 2024). One study demonstrated that the flagellin of *D. vulgaris* exacerbated ulcerative colitis in mice by activating the NAIP/NLRC4 inflammasome and inducing macrophage pyroptosis (An et al., 2023). Another study revealed that *D. vulgaris* or its flagellin interacted with LRRC19, rather than TLR5, to activate related signaling pathways, promoting the production of pro-inflammatory cytokines and aggravating ulcerative colitis (Xie et al., 2024). Furthermore, transplantation of *D. vulgaris* into healthy mice can induce intestinal inflammation, disrupt the intestinal barrier, and reduce short-chain fatty acid levels, thereby significantly exacerbating DSS-induced colitis (Huang et al., 2024). A previous study suggested that inhibiting *Desulfovibrio* could mitigate acute gastrointestinal inflammation in sepsis (Han C. et al., 2021). Additionally, *Desulfovibrio* has been reported to promote the production of LPS, a key component of Gram-negative bacterial outer membranes, which plays a crucial role in triggering acute inflammation and sepsis (Weglarz et al., 2003; Novakovic et al., 2016). Moreover, an increased abundance of *Desulfovibrio* has been positively correlated with elevated serum levels of AST and ALT, both indicators of liver injury (Cao et al., 2020). Although the role of *D. vulgaris* in various diseases has been preliminarily explored, studies on the specific role of *D. vulgaris* in sepsis are limited. The changes in its abundance within the gut microbiota of septic patients and its direct impact on disease progression remain unclear.

To address this issue, we established a murine sepsis model to explore the role of *D. vulgaris* in sepsis. We compared the abundance of *D. vulgaris* in the gut microbiota of septic mice and patients. To further investigate the impact of *D. vulgaris* on sepsis progression, we examined the inflammatory responses and oxidative stress across multiple organs. Our findings revealed a significant increase in *D. vulgaris* abundance in septic mice and patients. Notably, *D. vulgaris* exacerbated systemic inflammation by activating the mitogen-activated protein kinase (MAPK) and nuclear factor kappa B (NF-κB) signaling pathways in macrophages, contributing to multi-organ damage. Additionally, *D. vulgaris* promoted oxidative stress in various tissues, intensifying the pathological damage associated with sepsis. These results highlight the pivotal role of *D. vulgaris* in sepsis progression and suggest that targeting its growth or function could provide a novel therapeutic approach for alleviating sepsis-induced organ damage.

## Materials and methods

### Human samples

All human serum samples utilized in this study were procured from Nanfang Hospital. Patients diagnosed with sepsis in the ICU were selected based on the SEPSIS 3.0 criteria (Singer et al., 2016), while non-septic samples were collected from individuals undergoing trauma-related surgical procedures. Written informed consent was obtained from all participants prior to their inclusion. The study was approved by the Medical Ethics Committee of NanFang Hospital of Southern Medical University under the reference number NFEC-202403-K11. The baseline characteristics of the patients are summarized in Table 1.

**Table 1:** Baseline characteristics of patients.

Characteristic	Group		
	Overall *n* = 31	Non-sepsis *n* = 15	Sepsis *n* = 16
Age, years, median (IQR)	66 (49, 73)	52 (40, 57)	73 (69, 76)
Male	60 (47, 73)	47 (35, 54)	73 (71, 75)
Female	69 (56, 75)	58 (53, 72)	71 (69, 77)
Gender, *n* (%)			
Male	20 (64.5%)	10 (66.7%)	10 (62.5%)
Female	11 (35.5%)	5 (33.3%)	6 (37.5%)
Hospitalization days, median (IQR)	10 (6, 15)	13 (10, 18)	7 (5, 10)

### Mouse cecal ligation and puncture model

Male C57BL/6 mice (6–8 weeks old, 20–22 g) were obtained from Beijing Sibefur Laboratory Animal Technology Co., Ltd. Mice were housed under a 12-h light/dark cycle with free access to food and water at 22–24°C. Anesthesia was induced with isoflurane and confirmed by the loss of the righting reflex. The abdominal area was disinfected with 75% ethanol, and the fur was shaved. A midline incision (~2 cm) was made to expose the cecum, taking care not to damage the mesenteric vessels. The cecum was ligated ~1 cm from the tip using 4–0 silk and punctured once with an 18G needle at the distal portion (~3/4 from the tip). A small amount of cecal content was extruded to keep the puncture site open. The cecum was returned to the abdominal cavity, and the incision was closed in layers. Post-surgery, mice received saline (50 mL/kg) for resuscitation and were kept on a heated pad. Survival was monitored for 36 h, and Kaplan–Meier survival curves were generated. Tissue samples for multi-organ injury analysis were collected 6 h or 12 h after CLP. The animal study was approved by the Animal Ethics Committee of the Animal Experiment Center of Southern Medical University. All procedures were conducted in accordance with local legislation and institutional guidelines.

### Mouse endotoxemia model

Male C57BL/6 mice were intraperitoneally injected with 25 mg/kg lipopolysaccharide (LPS from *E. coli* O111:B4; Sigma, Cat# L2630) to induce endotoxemia. Cecal contents were harvested 12 h after LPS administration for further analysis.

### *Desulfovibrio vulgaris* culture

*Desulfovibrio vulgaris* (Cat# bio-092027) was obtained from Biobw. The strain was cultured anaerobically for 48 h on Columbia blood agar plates at 37°C. After incubation, bacterial colonies were scraped from the plates, centrifuged, and resuspended for enumeration. Mice were orally gavaged with *D. vulgaris* at a dose of 2 × 10^8^ CFU per mouse daily for 7 consecutive days. Following this treatment, CLP surgery was performed to induce sepsis.

### Bacterial DNA extraction

Approximately 20–30 mg of cecal contents were mixed with 500 μL of PBS containing 0.5% Tween 20, homogenized, and vortexed. The sample underwent three freeze–thaw cycles (−80°C for 10 min, 60°C for 5 min, repeated three times) to disrupt the cell walls. Then, 5 μL of proteinase K (20 mg/mL), 10 μL of RNase (0.1 mg/μL), and 25 μL of 10% SDS were added, and the mixture was incubated at 55°C for 1 h. After incubation, 75 μL of phenol was added and mixed, followed by an equal volume of PCI solution. The mixture was centrifuged at 13,000 RPM for 10 min at 4°C, and the supernatant was collected. The extraction was repeated with PCI solution and chloroform (1:1 ratio) with 3 M sodium acetate (1/10th volume), followed by centrifugation. To precipitate the DNA, 2.5 volumes of pre-chilled ethanol were added, and the mixture was centrifuged again. The DNA pellet was washed with 80% ethanol, air-dried, and resuspended in 100 μL ultrapure water. The DNA was stored at −20°C for further analysis.

### Desulfovibrio abundance analysis

The relative abundance of the *Desulfovibrio* genus and key species, including *D. vulgaris*, *D. piger*, *D. desulfuricans*, and *D. fairfieldensis*, was quantified using real-time quantitative PCR (qPCR). Specific primers targeting these taxa are listed in Table 2. qPCR reactions were performed in triplicate using a SYBR Green-based master mix (TOYOBO, Cat# QPK-201). The cycling conditions were optimized to ensure high specificity and amplification efficiency. Relative gene expression levels were calculated using the 2^−ΔΔCt^ method, with 16S rRNA serving as the internal control for normalization.

**Table 2:** Primers for *Desulfovibrio* abundance qPCR analysis.

	Forward primer (5′-3′)	Reverse primer (5′-3′)	References
*16S*	GTGSTGCAYGGYTGTCGTCA	ACGTCRTCCMCACCTTCCTC	Maeda et al. (2003)
*Desulfovibrio genus*	CCGTAGATATCTGGAGGAACATCAG	ACATCTAGCATCCATCGTTTACAGC	Fite et al. (2004)
*D. vulgaris*	GGCATCTGTAGACCTCCTTGTAGTC	TGTCGATCGTAGGTAGCAAATGGCG	An et al. (2023)
*D. piger*	GGAACTGCCCTTGATACTGC	CCGAGACATACATCCCGACA	This study
*D. intestinalis*	CACACTGGGACTGAAACACG	CGTCAATGCACCGCTGATTA	This study
*D. desulfuricans*	AACTTGAATCCGGGAGAGGG	GCATCCATCGTTTACAGCGT	This study
*D. fairfieldensis*	GTTTCGATCCAGTCCATGCC	GGCGGAAGTAAACGAAAGCA	This study

### Real-time quantitative PCR analysis

To evaluate the transcriptional levels of inflammatory cytokines and chemokines in mouse tissues, total RNA was isolated under RNase-free conditions using TRIzol Reagent (Invitrogen, Cat# 15596018). The RNA was then reverse-transcribed into complementary DNA (cDNA) using a reverse transcription kit (TOYOBO, Cat# FSQ-101). qPCR was performed on a Roche LightCycler^®^ 96 Real-Time PCR System with SYBR Green as the fluorescent dye. Gene-specific primers for inflammatory cytokines and chemokines were designed and are listed in Table 3. The 18S ribosomal RNA (18S rRNA) served as an internal reference gene for normalization. Relative gene expression levels were calculated using the 2^−ΔΔCt^ method.

**Table 3:** Primers for cytokine and chemokine qPCR analysis.

	Forward primer (5′-3′)	Reverse primer (5′-3′)
*18S*	AGTCCCTGCCCTTTGTACACA	CGATCCGAGGGCCTCACTA
*Tnf-α*	CAGGCGGTGCCTATGTCTC	CGATCACCCCGAAGTTCAGTAG
*Il-6*	CTGCAAGAGACTTCCATCCAG	AGTGGTATAGACAGGTCTGTTGG
*Il-1β*	GAAATGCCACCTTTTGACAGTG	TGGATGCTCTCATCAGGACAG
*Ccl2*	TAAAAACCTGGATCGGAACCAAA	GCATTAGCTTCAGATTTACGGGT
*Ccl3*	TGTACCATGACACTCTGCAAC	CAACGATGAATTGGCGTGGAA
*Cxcl2*	CCAACCACCAGGCTACAGG	GCGTCACACTCAAGCTCTG
*Cxcl10*	CCAAGTGCTGCCGTCATTTTC	GGCTCGCAGGGATGATTTCAA
*Zo-1*	GTTGGTACGGTGCCCTGAAAGA	GCTGACAGGTAGGACAGACGAT
*Occludin*	CATTTATGATGAACAGCCCC	GGACTGTCAACTCTTTCCGC
*Claudin-1*	GGACTGTGGATGTCCTGCGTTT	GCCAATTACCATCAAGGCTCGG

### ALT and AST assays

Serum ALT and AST levels were measured by a kinetic method using commercial assay kits (Nanjing Jiancheng Bioengineering Institute; ALT: Cat# C009-3-1; AST: Cat# C010-3-1) following the manufacturer’s instructions. Peripheral blood was collected and centrifuged at 12,000 rpm for 15 min at 4°C to isolate serum. In a 96-well plate, 200 μL of Reagent 1, 50 μL of Reagent 2, and 10 μL of serum were mixed per well. The absorbance at 340 nm was monitored at both time points of 0 and 1 min at a temperature of 37°C using a microplate reader (Epoch, BioTek). The activities of ALT and AST were then calculated based on the formulas according to the manufacturer.

### Serum creatinine measurement

Serum creatinine (Cr) levels were determined using a creatinine assay kit (Nanjing Jiancheng Bioengineering Institute, Cat# C011-2-1) based on the sarcosine oxidase method. In a 96-well plate, 6 μL of serum samples, 6 μL of creatinine standard solution (442 μmol/L), and 6 μL of double-distilled water were added to the test wells, standard wells, and blank wells, respectively. Subsequently, 180 μL of Enzyme Solution A was added to each well. The plate was incubated at 37°C for 5 min, and absorbance at 546 nm (A1) was recorded using a microplate reader. Then, 60 μL of Enzyme Solution B was added to each well, followed by another 5-min incubation at 37°C. Absorbance at 546 nm (A2) was recorded, and creatinine levels were calculated according to the kit’s formula.

### Blood urea nitrogen measurement

Blood urea nitrogen (BUN) was measured using a urea nitrogen assay kit (Cat# C013-2-1, Nanjing Jiancheng Bioengineering Institute) via the urease method. In brief, 20 μL of serum samples, 20 μL of urea nitrogen standard solution (10 mM), and 20 μL of double-distilled water were added to the test wells, standard wells, and blank wells, respectively. Each well was supplemented with 250 μL of buffer enzyme solution, mixed by vortexing for 10 s, and incubated in a water bath at 37°C for 10 min. Next, 1 mL of color reagent and 1 mL of alkaline sodium hypochlorite solution were added, vortexed for 10 s to ensure complete mixing, and incubated again at 37°C for 10 min. Subsequently, 200 μL of the solution was transferred to a 96-well plate, and the absorbance was measured at 640 nm using a microplate reader. BUN levels were calculated according to the kit’s formula.

### Measurement of GSH and antioxidant enzyme activities

For the determination of GSH and antioxidant enzyme activities, 100 mg of fresh tissue samples were homogenized in 900 μL of PBS (pH 7.4, 0.1 M). The homogenate was then centrifuged at 3,500 × *g* for 10 min, and the resulting supernatant was used to measure the activities of GSH and antioxidant enzyme activities including superoxide dismutase (SOD) and catalase (CAT) using commercially available assay kits (Nanjing Jiancheng Bioengineering Institute). Protein concentration in the supernatant was quantified using a Pierce BCA Protein Assay Kit (Pierce, Cat# 23225).

The GSH content was determined using the Glutathione Assay Kit (Cat# A006-2-1) according to the DTNB [5,5′-dithiobis (2-nitrobenzoic acid)] method. Absorbance was measured at 405 nm, and the GSH content was expressed as μmol per g protein.

CAT activity was assessed using the CAT Assay Kit (Cat# A007-1-1), and results were expressed as U per mg protein. One unit of CAT activity is defined as the amount of enzyme that decomposes 1 μmol of H₂O₂ per minute per mg of fresh sample at 37°C.

SOD activity was measured using the SOD Assay Kit (Cat# A001-1-2) and expressed as U per mg protein. One unit of SOD activity corresponds to the amount of extract that causes 50% inhibition in the reduction of xanthine, as monitored at 550 nm.

### Hematoxylin and eosin staining

Mouse liver, kidney, and lung tissues were fixed in 4% paraformaldehyde for 24 h, followed by dehydration, clearing, paraffin embedding, and sectioning. The embedded tissues were cut into 5 μm sections, baked at 60°C for 1 h, and stored for later use. For staining, sections were deparaffinized in xylene (twice for 10 min each), then sequentially rehydrated through a graded ethanol series (100, 95, 90, and 80%, each for 2 min) before rinsing in distilled water. Hematoxylin staining was performed for 8 min, followed by rinsing with running tap water for 10 min. Sections were then differentiated in 1% hydrochloric acid alcohol for 10 s and rinsed with running tap water for 10 min to restore the blue color. Eosin staining was performed for 3 min, followed by sequential dehydration in 80, 90, 95, and 100% ethanol, and clearing in xylene (twice for 5 min each). Histological evaluation was conducted by capturing at least six random fields per H&E-stained slide under a light microscope. Liver, kidney, and lung injuries were assessed and scored based on histopathological criteria described in a previous study published by our research group (Chen et al., 2023). In brief, liver injury was assessed based on necrosis, inflammation, ballooning degeneration, and hepatic cord disruption, with each parameter scored from 0 to 3. Lung injury was evaluated based on alveolar congestion, hemorrhage, inflammatory cell infiltration, and alveolar wall thickening, with each parameter scored from 0 to 3. Kidney injury was assessed based on the extent of tubular and glomerular damage, with a scoring range of 0 to 5.

### Measurement of ROS production

ROS production in tissues was assessed using the dihydroethidium (DHE) oxidation assay. Frozen liver, lung, and kidney sections were incubated with 5 μM DHE (Thermo Scientific, Cat# D23107) at 37°C for 20 min. After incubation, the ROS levels were observed using a Leica microscope (Leica DMi8), and the average fluorescence intensity was measured and analyzed using ImageJ software.

### TUNEL staining

To evaluate cell death in the liver, kidney, and lung tissues of CLP mice, paraffin-embedded tissue sections were subjected to terminal deoxynucleotidyl transferase dUTP nick end labeling (TUNEL) staining using a commercial kit (KeyGEN BioTECH, Cat# KGA1405-100) according to the manufacturer’s instructions. Briefly, tissue sections were deparaffinized in xylene, rehydrated through a graded ethanol series, and rinsed with PBS. Permeabilization was performed by incubating the sections with Proteinase K (20 μg/mL) at 37°C for 30 min, followed by PBS washes. The TdT enzyme reaction mixture was prepared by combining Equilibration Buffer, biotin-11-dUTP, and TdT Enzyme, and 50 μL of the mixture was applied to each section. The slides were incubated in a humidified chamber at 37°C in the dark for 60 min. After washing with PBS, a Streptavidin-TRITC labeling solution was applied, and the slides were incubated at 37°C in the dark for 30 min. Following another PBS wash, nuclei were counterstained with DAPI (1 μg/mL) for 10 min at room temperature. Excess DAPI was removed by rinsing with PBS, and the sections were mounted with an anti-fade medium and coverslipped. TUNEL-positive cells (apoptotic cells) were identified by red fluorescence under a fluorescence microscope, while DAPI stained all nuclei blue. The number of TUNEL-positive cells was quantified using image analysis software.

### ELISA

Enzyme-linked immunosorbent assay (ELISA) was performed to determine the serum concentrations of TNF-*α*, IL-1β, and IL-6 using commercial ELISA kits from Neobioscience according to the manufacturer’s instructions (TNF-α: Cat# EMC102a, IL-1β: Cat# EMC001b, IL-6: Cat# EMC004). Briefly, serum samples were appropriately diluted. A 100 μL aliquot of each diluted sample or standard was added and incubated at 37°C for 90 min. Following incubation, the plate was washed, and a biotinylated detection antibody solution (100 μL) was added to each well and incubated at 37°C for 60 min. After washing, 100 μL of enzyme-conjugated streptavidin was added to each well and incubated at 37°C for 30 min. The plate was washed again, and 100 μL of TMB substrate solution was added to each well. The reaction was allowed to develop, then stopped using the stop solution. The absorbance was measured at 450 nm using a microplate reader. Cytokine concentrations in the samples were determined by comparing the absorbance values to the standard curve generated using the provided standards.

### Peritoneal macrophage sorting

Peritoneal lavage fluid from septic mice was passed through a 70 μm nylon mesh and centrifuged. The cell pellet was resuspended in MACS buffer (PBS with 0.5% BSA and 2 mM EDTA). To label macrophages, 10 μL of F4/80 MicroBeads antibody (Miltenyi Biotec, Cat# 130–110-443) was added, followed by a 20-min incubation at 4°C. F4/80-positive macrophages were then isolated using LS columns (Miltenyi Biotec) according to the manufacturer’s instructions.

### Western blot analysis

Total protein was extracted using RIPA buffer (Beyotime, Cat# P0013C), and protein concentrations were determined using a Pierce BCA Protein Assay Kit. Equal amounts of protein were separated by 10% SDS-PAGE and transferred onto nitrocellulose membranes. The membranes were blocked with 5% BSA and incubated overnight at 4°C with primary antibodies against SAPK/JNK (1:1,000, Proteintech, Cat# 66210-1-Ig), phospho-SAPK/JNK (1:1,000, CST, Cat# 9255S), ERK1/2 (1:1,000, CST, Cat# 4695S), phospho-ERK1/2 (1:1,000, CST, Cat# 9101S), NF-κB P65 (1:1,000, CST, Cat# 8242S), phospho-NF-κB P65 (1:1,000, CST, Cat# 3033S), P38 MAPK (1:1,000, Proteintech, Cat# 14064-1-AP), and phospho-P38 MAPK (1:1,000, CST, Cat# 9211S). ZO-1 (1:1,000, Affinity, Cat# AF5145), Occludin (1:1,000, Affinity, Cat# DF7504), and Claudin-1 (1:1,000, Affinity, Cat# AF0127). After washing, the membranes were incubated with HRP-conjugated secondary antibodies (RayBiotech; Goat anti-Mouse IgG(H + L)-HRP: Cat# RM3001, Goat anti-Rabbit IgG(H + L)-HRP: Cat# RM3002). Protein bands were visualized using an enhanced chemiluminescence system (Bio-Rad) and quantified with ImageJ software.

### Immunohistochemistry

Paraffin-embedded tissue sections were deparaffinized and rehydrated, followed by antigen retrieval in citrate buffer using a microwave for 15 min. After cooling to room temperature, endogenous peroxidase activity was blocked with 3% hydrogen peroxide for 10 min, and nonspecific binding was blocked with 3% BSA for 30 min. Sections were incubated overnight at 4°C with a primary antibody against CD11b (1:100, Servicebio, Cat# GB11058). The following day, sections were washed with PBS and incubated with a secondary antibody at room temperature for 1 h. The signal was developed using 3,3′-diaminobenzidine (DAB), followed by hematoxylin counterstaining. Images were acquired using a microscope, capturing at least five fields of view per section. Quantification of CD11b positive area was performed using the IHC profile plugin in ImageJ software.

### Statistical analysis

All statistical analyses were performed using GraphPad Prism software. Data were first tested for normality using the Shapiro–Wilk test and for homogeneity of variances using Levene’s test. If the data met the assumptions of normality and homogeneity of variance, comparisons between the two groups were conducted using an unpaired Student’s t-test. Survival analyses were conducted using the Kaplan–Meier method with log-rank tests. Data were expressed as mean ± SEM. Statistical significance was indicated as follows: *p < 0.05, **p < 0.01, ***p < 0.001, and ****p < 0.0001.

## Results

### Increased abundance of *Desulfovibrio vulgaris* in the gut microbiota of septic mice and patients

**Figure 1:**
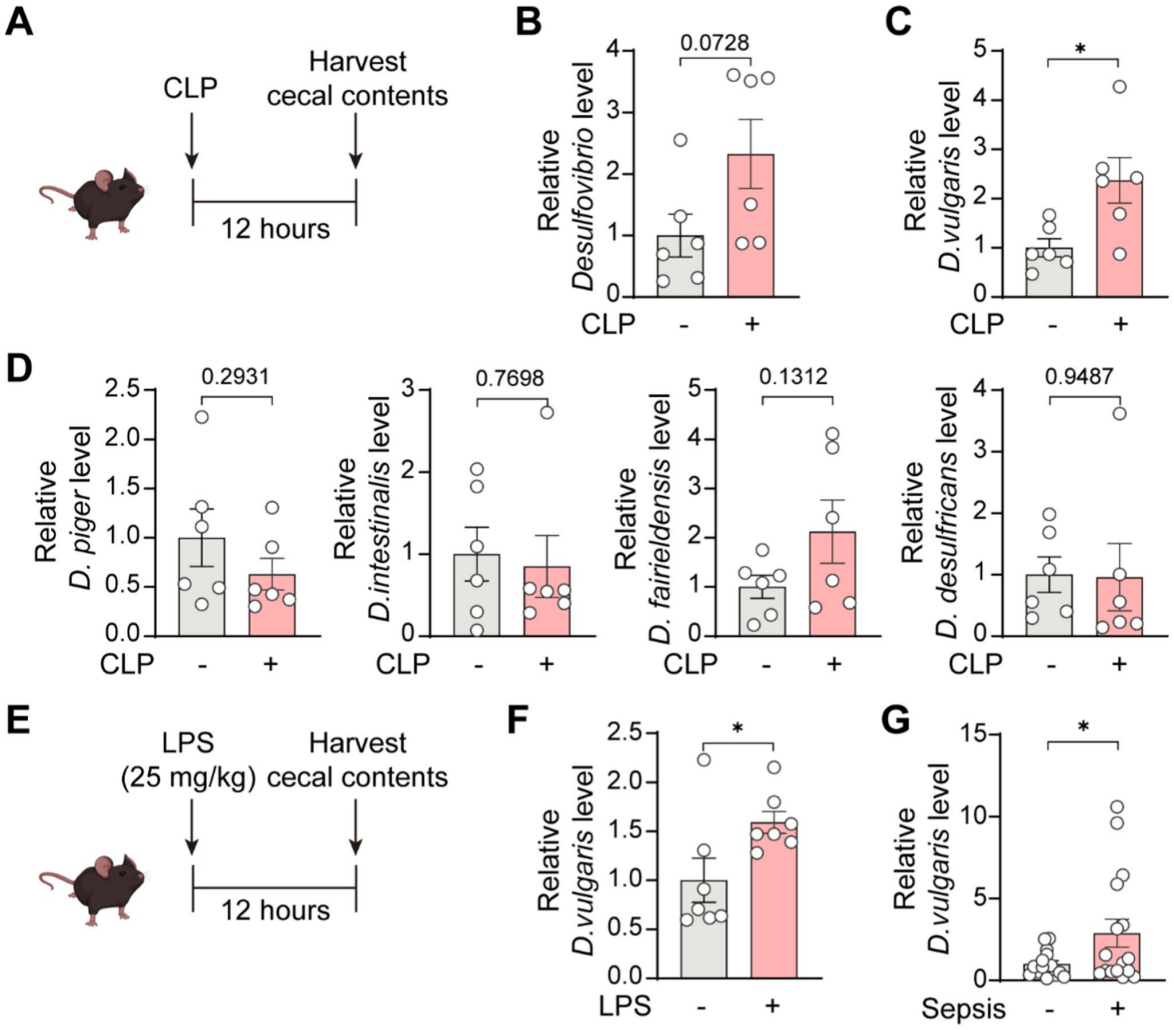
Increased abundance of *D. vulgaris* in the gut microbiota of septic mice and patients. **(A)** Cecal contents were collected from septic mice 12 h post-CLP surgery, and bacterial DNA was extracted. **(B)** qPCR analysis of the abundance of *Desulfovibrio genus* in the cecal microbiota of septic mice. *n* = 6. **(C)** qPCR analysis revealing increased *D. vulgaris* abundance in septic mouse cecal microbiota. *n* = 6. **(D)** qPCR analysis of the abundance of *D. piger*, *D. intestinalis*, *D. fairfieldensis*, and *D. desulfuricans* in the cecal microbiota of septic mice. *n* = 6. **(E)** Cecal contents were collected 12 h post-intraperitoneal LPS injection (25 mg/kg), with bacterial DNA extraction. **(F)** qPCR analysis showing increased *D. vulgaris* abundance in the cecal microbiota of endotoxemic mice. *n* = 7. **(G)** Comparison of *D. vulgaris* abundance in the cecal microbiota of septic versus non-septic patients. *n* = 15–16. Data are presented as mean ± SEM. Statistical comparisons were performed using two-tailed unpaired Student’s t-test. *p < 0.05.

*Desulfovibrio* species are Gram-negative, anaerobic bacteria that are prevalent in the human gut microbiome (Singh et al., 2023). We collected cecal contents from septic mice 12 h after CLP surgery and extracted bacterial DNA (Figure 1A). Then, we assessed the abundance of *Desulfovibrio* species and several of its strains. The results showed a trend toward increased *Desulfovibrio* abundance in the cecum of septic mice, although this did not reach statistical significance (Figure 1B). Notably, *D. vulgaris* abundance in the cecum of septic mice was significantly elevated (Figure 1C), whereas the abundance of other *Desulfovibrio* species, including *D. piger*, *D. intestinalis*, *D. fairfieldensis*, and *D. desulfuricans*, remained unchanged (Figure 1D). To confirm whether this effect was observed in endotoxemia, we induced endotoxemia in mice by intraperitoneal injection of lipopolysaccharide (LPS; 25 mg/kg) and collected cecal contents for DNA extraction 12 h later (Figure 1E). qPCR analysis revealed a significant increase in *D. vulgaris* abundance in the cecum of endotoxemic mice (Figure 1F). Furthermore, in sepsis patients, the abundance of *D. vulgaris* was significantly higher compared to non-septic controls (Figure 1G). Collectively, these findings indicate a significant increase in *D. vulgaris* abundance in the gut microbiota of both septic mice and patients, suggesting that *D. vulgaris* may play a pivotal role in the pathogenesis of sepsis.

### *Desulfovibrio vulgaris* exacerbates mortality and organ damage in septic mice

**Figure 2:**
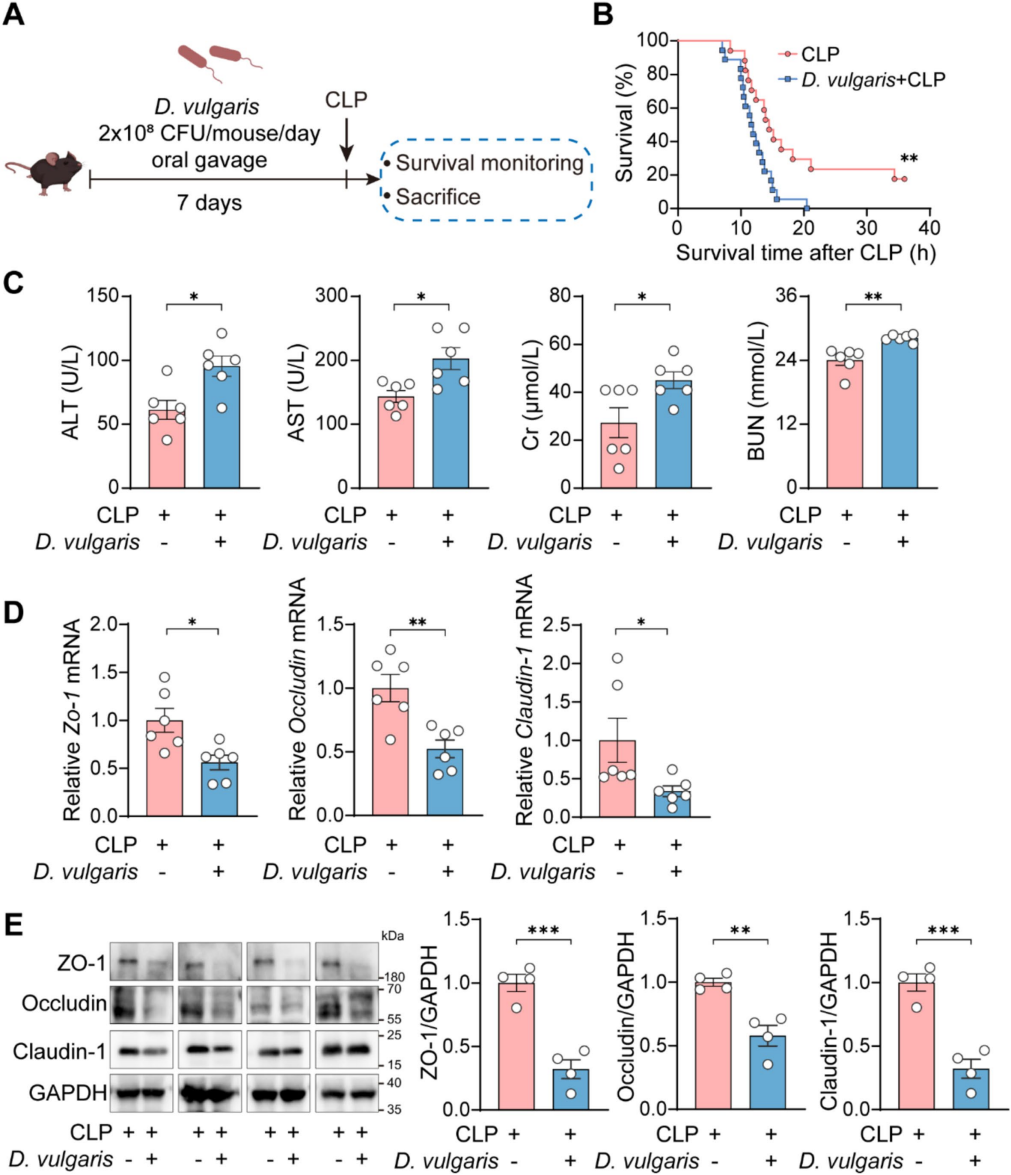
*D. vulgaris* exacerbates mortality and organ dysfunction in septic mice. **(A)** Experimental design for *D. vulgaris* administration: mice were gavaged with *D. vulgaris* (2 × 10^8^ CFU/mouse) daily for 7 days before CLP surgery. Survival was monitored for 36 h, and tissues were collected 12 h post-CLP. **(B)** Survival rates of *D. vulgaris*-treated mice after CLP. *n* = 17–18. **(C)** Serum ALT, AST, Cr, and BUN levels in septic mice after *D. vulgaris* gavage. *n* = 6. **(D)** The relative mRNA levels of *Zo-1*, *Occludin*, and *Claudin-1* in the colon. *n* = 6. **(E)** The protein expression levels of ZO-1, Occludin, and Claudin-1 in the colon. *n* = 4. Data are presented as mean ± SEM. Survival rates were analyzed using the Kaplan–Meier method with the log-rank test **(B)**. Statistical comparisons were performed using two-tailed unpaired Student’s t-tests **(C–E)**. *p < 0.05, **p < 0.01, ***p < 0.001.

**Figure 3:**
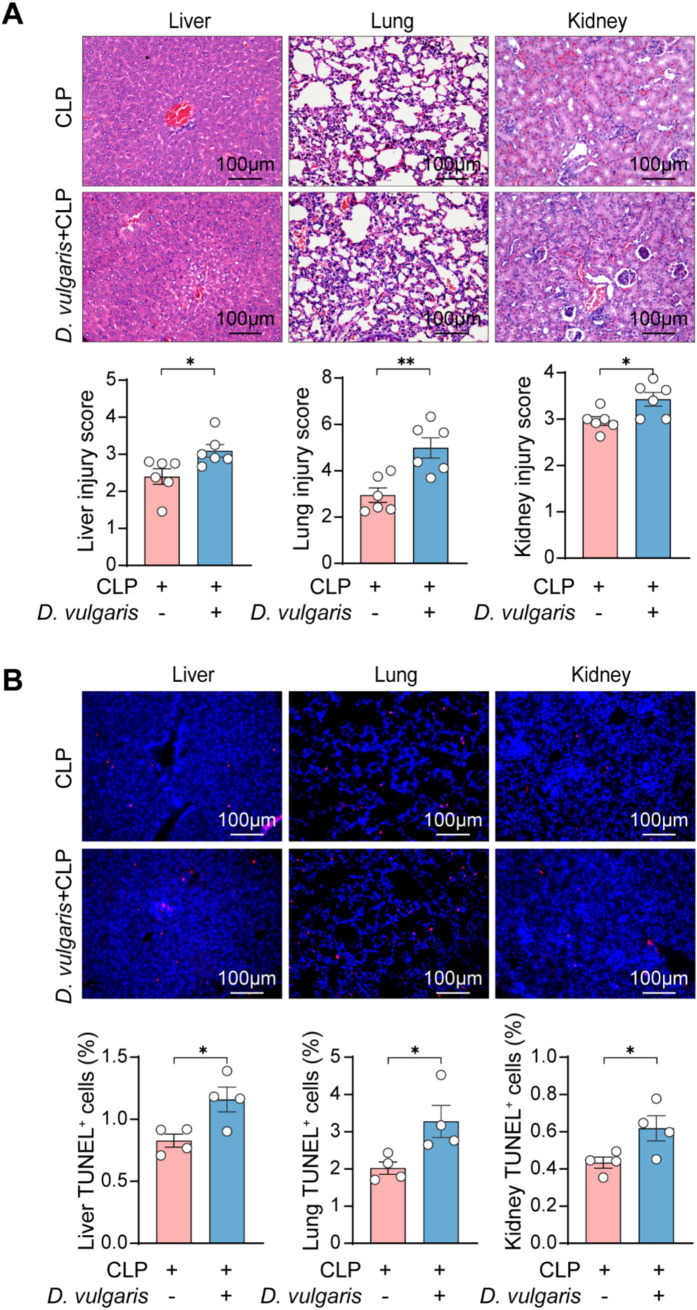
*D. vulgaris* promotes multi-organ pathological damage in septic mice. **(A)** Representative H&E-stained histological images and quantitative analysis of liver, kidney, and lung tissue damage in septic mice after *D. vulgaris* administration. *n* = 6. **(B)** TUNEL staining (red: TUNEL-positive cells, blue: cell nuclei) showing apoptosis in liver, kidney, and lung tissues of septic mice after *D. vulgaris* gavage. *n* = 4. Data are presented as mean ± SEM. Statistical comparisons were performed using two-tailed unpaired Student’s t-test. *p < 0.05, **p < 0.01. Scale bars, 100 μm.

To further explore the effects of *D. vulgaris* in sepsis, mice were gavaged with *D. vulgaris* (2 × 10^8^ CFU/mouse/day) for 7 consecutive days, followed by CLP surgery. Survival rates were monitored for 36 h post-surgery, and tissues were collected for analysis (Figure 2A). *D. vulgaris* successfully colonized the gut, as evidenced by its significantly increased abundance in mouse feces (Supplementary Figure S1A). The results showed that *D. vulgaris* significantly reduced the survival rate of mice with sepsis compared to controls (Figure 2B), indicating that *D. vulgaris* exacerbates sepsis-induced mortality. Additionally, *D. vulgaris* treatment markedly elevated serum levels of ALT, AST, Cr, and BUN in septic mice (Figure 2C), suggesting damage to the liver and kidney tissues. However, *D. vulgaris* did not elevate ALT, AST, Cr, or BUN levels in healthy mice (Supplementary Figure S1B). Furthermore, *D. vulgaris* aggravated intestinal mucosal barrier damage in septic mice (Figures 2D,E), whereas it had no impact on the intestinal mucosal barrier in healthy mice (Supplementary Figure S1C). To investigate the direct impact of *D. vulgaris* on organ tissues, histological analysis was performed. Treatment with *D. vulgaris* markedly aggravated pathological damage in the liver, kidney, and lung tissues of septic mice. (Figure 3A). However, the proliferation of *D. vulgaris* did not cause pathological damage in the tissues of healthy mice (Supplementary Figure S2). TUNEL staining revealed a significant increase in apoptosis in these organs following *D. vulgaris* treatment (Figure 3B). These results suggest that *D. vulgaris* exacerbates multi-organ pathological damage in sepsis through induction of tissue injury and cellular apoptosis.

### *Desulfovibrio vulgaris* enhances inflammatory responses in septic mice

**Figure 4:**
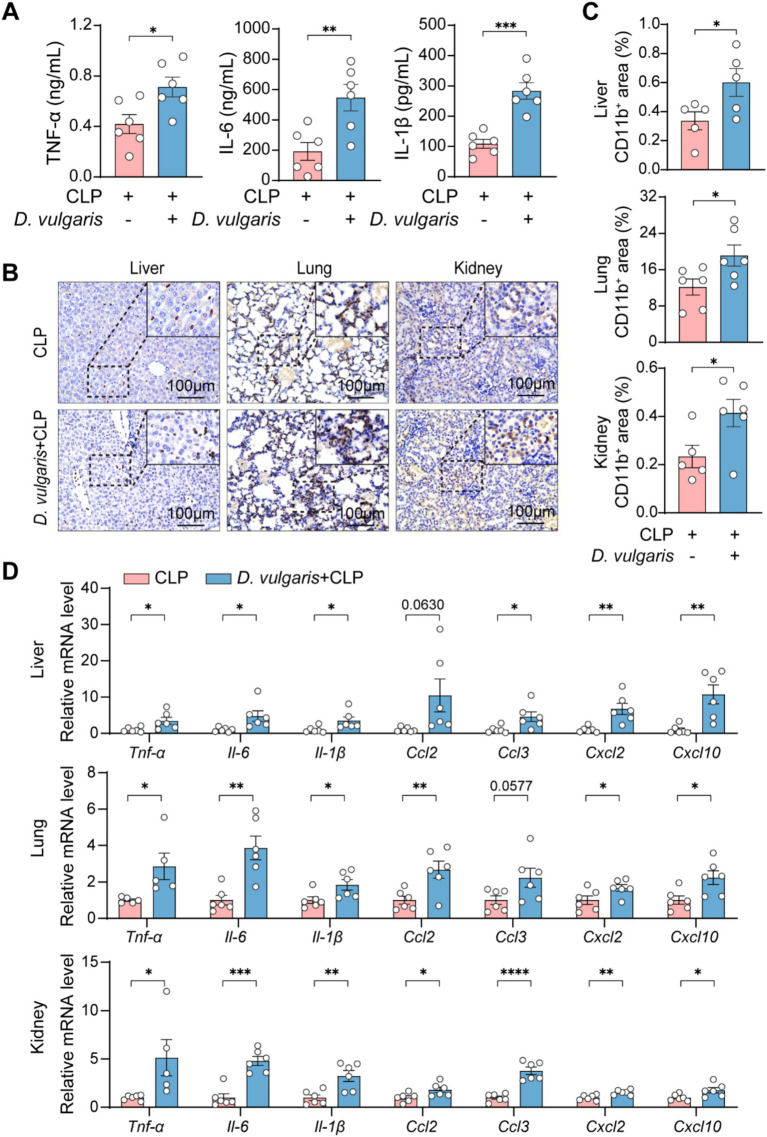
*D. vulgaris* induces inflammation in septic mice. **(A)** ELISA measurement of TNF-*α*, IL-1β, and IL-6 levels in the serum of *D. vulgaris*-treated septic mice. *n* = 6. **(B,C)** Immunohistochemical staining showing CD11b^+^ cell infiltration in liver, kidney, and lung tissues. *n* = 5–6. **(D)** qPCR analysis of inflammatory cytokines and chemokines in liver, kidney, and lung tissues. Data are presented as mean ± SEM. Statistical comparisons were performed using two-tailed unpaired Student’s t-test. *p < 0.05, **p < 0.01, ***p < 0.001, ****p < 0.0001. Scale bars, 100 μm.

To examine the effect of *D. vulgaris* on inflammation, we measured levels of inflammatory cytokines in both serum and tissues. ELISA analysis showed that *D. vulgaris* treatment significantly elevated serum levels of the pro-inflammatory cytokines TNF-*α*, IL-1β, and IL-6 in septic mice (Figure 4A). Immunohistochemical analysis further demonstrated a marked increase in the infiltration of CD11b^+^ cells in the liver, kidneys, and lungs of septic mice treated with *D. vulgaris* (Figures 4B,C). Additionally, qPCR analysis revealed that *D. vulgaris* treatment significantly upregulated the transcriptional levels of inflammatory cytokines and chemokines in these organs (Figure 4D). These findings collectively suggest that *D. vulgaris* treatment exacerbates inflammation in septic mice, as evidenced by increased levels of pro-inflammatory cytokines, enhanced immune cell infiltration, and elevated gene expression of inflammatory mediators in key organs.

### *Desulfovibrio vulgaris* promotes the activation of MAPK and NF-κB pathways in septic mice

**Figure 5:**
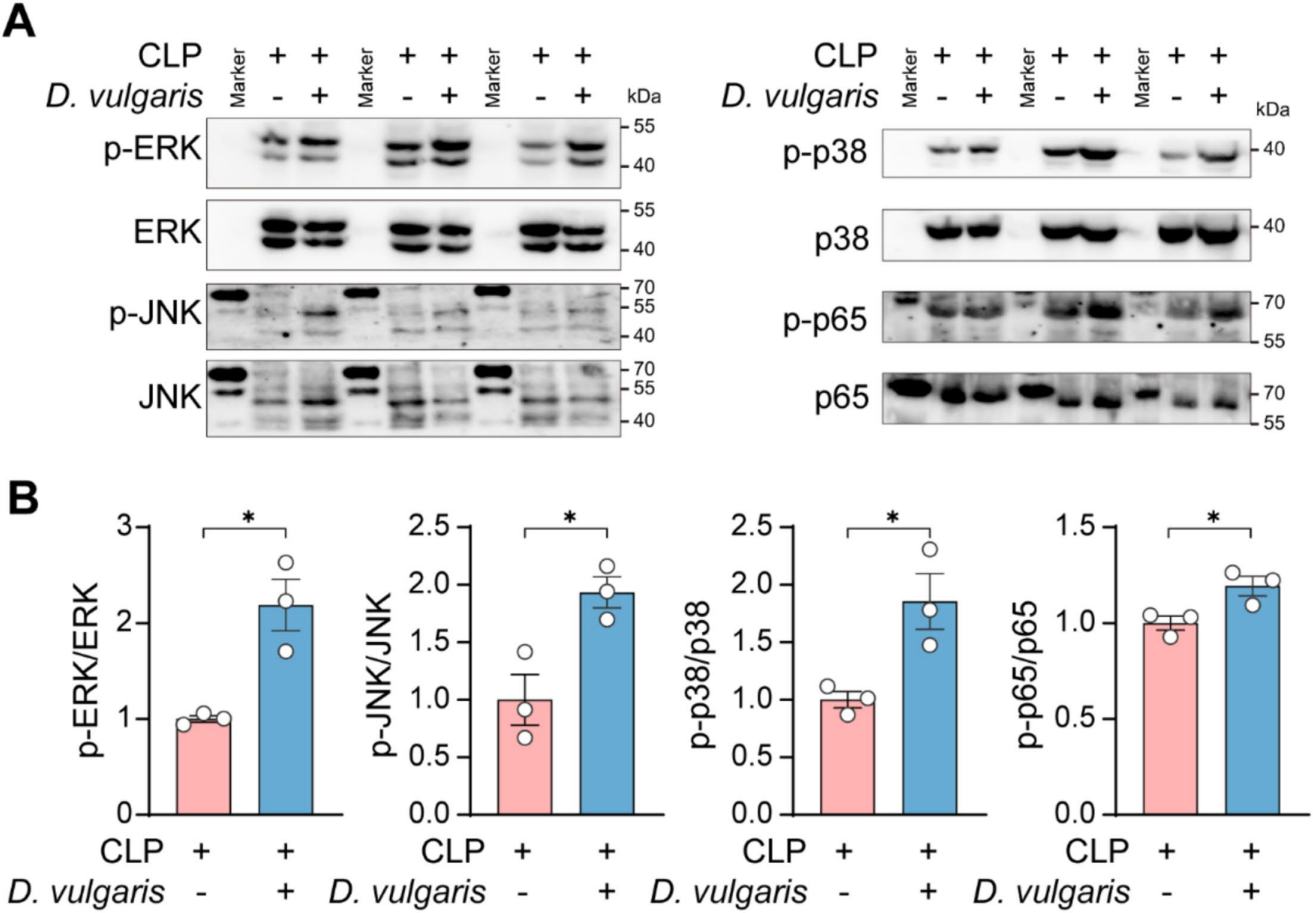
*D. vulgaris* promotes the activation of MAPK and NF-κB pathways in peritoneal macrophages of septic mice. **(A,B)** Western blot analysis of phosphorylated MAPK and NF-κB in peritoneal macrophages isolated from septic mice treated with *D. vulgaris*. *n* = 3. Data are presented as mean ± SEM. Statistical comparisons were performed using two-tailed unpaired Student’s t-test. *p < 0.05.

To investigate the molecular mechanisms of *D. vulgaris*-induced inflammation, we focused on key signaling pathways involved in immune activation. The MAPK and NF-κB pathways are central regulators of inflammation and are commonly activated in conditions like sepsis (Gottschalk et al., 2016; Zhang and Ning, 2021). MAPK signaling regulates gene expression, cell survival, and cytokine production and the MAPK family includes three major types of kinases: ERK (Extracellular signal-Regulated Kinase), JNK (c-Jun N-terminal Kinase), and p38 MAPK (Cargnello and Roux, 2011). NF-κB, a transcription factor family, controls immune responses and pro-inflammatory cytokine expression (Guo et al., 2024). Both pathways are activated by external stimuli, such as bacteria, virus, or injury (Rahman and McFadden, 2011; Dong et al., 2015; Lane et al., 2019; Liu S. Q. et al., 2020; Guo et al., 2023), and can amplify inflammatory responses by regulating each other’s effects (Nyati et al., 2017; Bansal et al., 2022). Given their critical roles in inflammation, we specifically investigated the effects of *D. vulgaris* on peritoneal macrophages from septic mice. Western blot analysis revealed significant phosphorylation of JNK, ERK, and p38 MAPK, as well as pronounced phosphorylation of NF-κB p65 in peritoneal macrophages from septic mice following *D. vulgaris* treatment (Figures 5A,B). These findings suggest that *D. vulgaris* induces inflammation through the activation of both MAPK and NF-κB signaling pathways, potentially driving its pro-inflammatory effects.

### *Desulfovibrio vulgaris* exacerbates oxidative stress injury in sepsis mice

**Figure 6:**
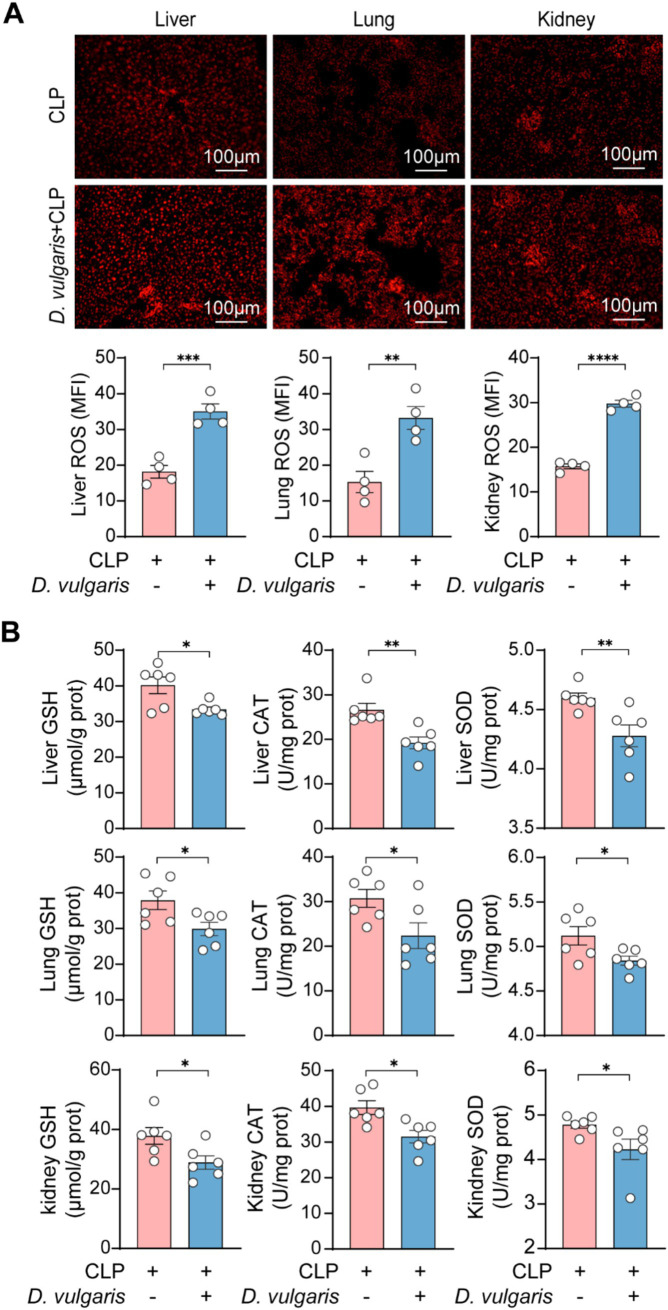
*D. vulgaris* induces oxidative stress in septic mice. **(A)** ROS levels in liver, kidney, and lung tissues of septic mice treated with *D. vulgaris*. *n* = 4. **(B)** Antioxidant levels (GSH, CAT, and SOD) in liver, kidney, and lung tissues of septic mice. *n* = 6. Data are presented as mean ± SEM. Statistical comparisons were performed using two-tailed unpaired Student’s t-test. *p < 0.05, **p < 0.01, ***p < 0.001, ****p < 0.0001. Scale bars, 100 μm.

Oxidative stress plays a central role in the pathophysiology of sepsis, driving inflammatory responses and contributing to organ dysfunction (Victor et al., 2009; McCreath et al., 2016). To assess the impact of *D. vulgaris* on oxidative stress during sepsis, we measured ROS levels in the liver, kidney, and lung tissues of septic mice. *D. vulgaris* treatment resulted in a significant elevation of ROS in all three tissues (Figure 6A), indicating a marked exacerbation of oxidative stress. Concomitantly, we observed a significant reduction in the levels of key antioxidant enzymes, including GSH, CAT, and SOD (Figure 6B), which are critical for maintaining redox homeostasis. These findings suggest that *D. vulgaris* disrupts the delicate balance between ROS and antioxidant defenses, thereby amplifying oxidative damage in septic tissues. This redox imbalance may further exacerbate tissue injury, promoting the progression of sepsis-related organ dysfunction. Collectively, our data underscore the pivotal role of oxidative stress in *D. vulgaris*-mediated exacerbation of sepsis pathophysiology and suggest potential therapeutic strategies targeting oxidative pathways to mitigate sepsis-associated organ damage.

**Figure 7:**
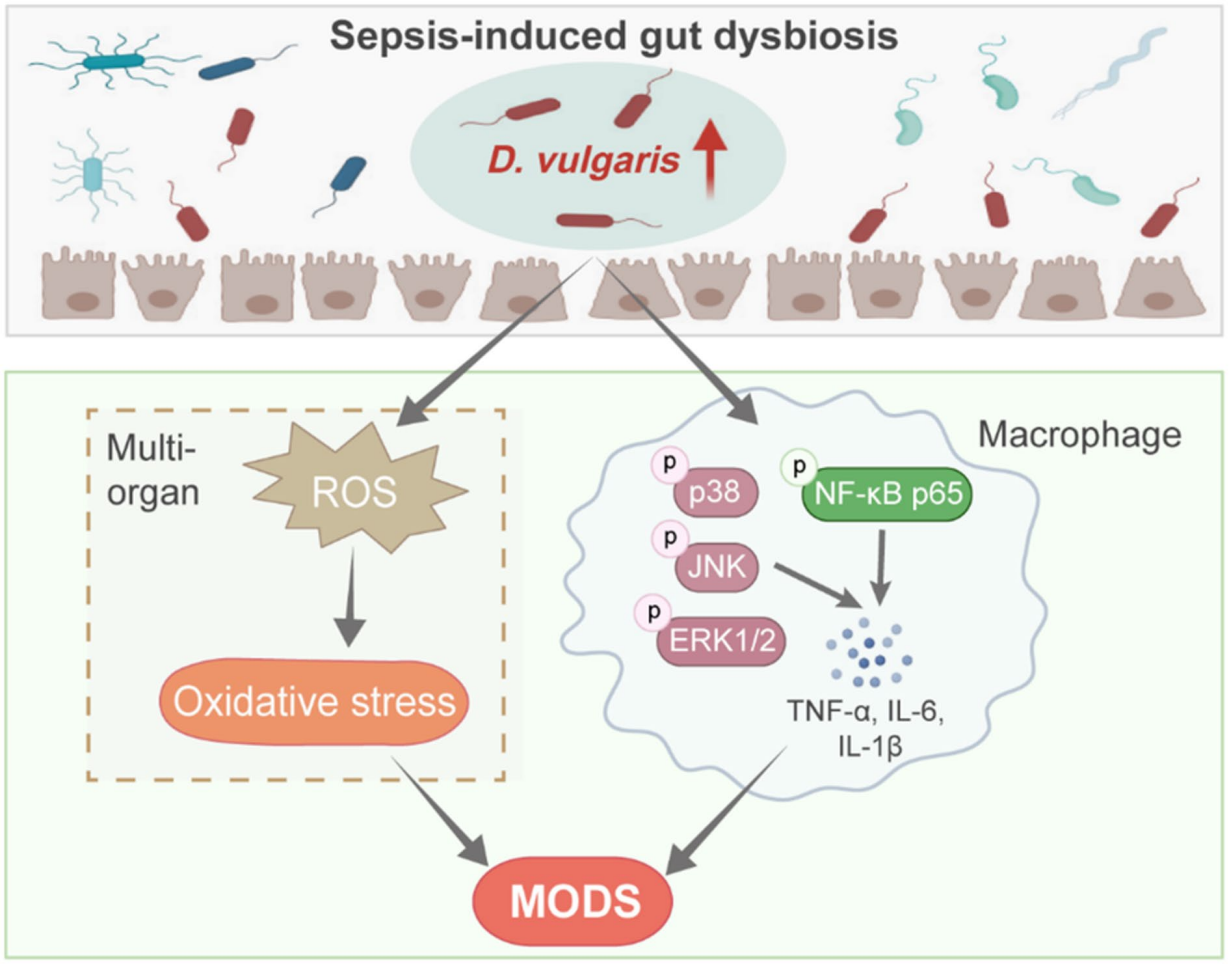
*D. vulgaris* promotes inflammation and oxidative stress in septic mice. Model illustrating the effects of increased *D. vulgaris* abundance in the gut during sepsis, leading to activated macrophage MAPK and NF-κB pathways, heightened inflammation, and increased oxidative stress in multiple organs.

Based on our findings, we propose the following model (Figure 7): Sepsis-induced dysbiosis increases the abundance of *D. vulgaris* in the gut. This shift activates MAPK and NF-κB signaling pathways in macrophages, leading to enhanced inflammation. Additionally, *D. vulgaris* promotes the production of ROS, exacerbating oxidative stress. Together, these processes worsen sepsis-related organ damage, increase tissue injury, and contribute to higher mortality.

## Discussion

The gut microbiota plays a central role in regulating immune responses and has recently been identified as a key factor in the pathogenesis of sepsis. Dysbiosis of the gut microbiota is widely recognized as a key pathological mechanism in sepsis (Haak and Wiersinga, 2017). Recent studies have shown that the gut microbiome not only plays a crucial role in immune system modulation but also significantly influences the progression of sepsis (Sun et al., 2022). Previous studies have shown that gut microbiota dysbiosis contributes to sepsis-induced liver injury (Gong et al., 2019), highlighting its significant impact on organ function and disease progression.

In our study, we observed an increase in the abundance of *Desulfovibrio* species, as a conditional pathogenic bacterium, in the cecum of septic mice. This finding suggests that *Desulfovibrio* may be in a delicate regulatory state within the gut microbiota, with its abundance potentially influenced by specific pathological conditions. The rise in *Desulfovibrio* abundance may be associated with gut microbiota dysbiosis, diet (high-fat, low-fiber), antibiotic use, and increased intestinal sulfur compounds (Han C. et al., 2021; Singh et al., 2023; Zhou H. et al., 2024). Previous studies show that *Desulfovibrio* displays variable behavior across different disease models, including alcohol-induced liver injury, inflammatory bowel disease, ulcerative colitis, liver fibrosis, colorectal cancer, and stroke (Loubinoux et al., 2002; Rowan et al., 2010; Yang et al., 2019; Wang et al., 2021; Li et al., 2024; Qi et al., 2024; Zhang et al., 2024). These observations reflect the complex role of *Desulfovibrio* in host–microbe interactions across different pathological contexts. Additionally, previous studies have linked *Desulfovibrio* species such as *D. fairfieldensis* and *D. desulfuricans* to the onset of bacteremia (Pimentel and Chan, 2007; Urata et al., 2008; Yamaizumi et al., 2024). These findings support the notion that *Desulfovibrio* acts as a potential pathogenic factor during sepsis.

Although the overall abundance of *Desulfovibrio* did not show significant changes in our sepsis models, we observed a marked increase in *D. vulgaris* abundance in the cecum of CLP-induced septic mice, which was confirmed in the LPS-induced endotoxemia model. Previous research has shown that *D. vulgaris* proliferation exacerbates intestinal inflammation in ulcerative colitis, with the extent of proliferation positively correlating with the severity of intestinal disease (Rowan et al., 2010; An et al., 2023; Xie et al., 2024). This reinforces the key role of *D. vulgaris* in gut barrier function, immune responses, and disease progression.

In our septic mouse model, *D. vulgaris*-treated mice had significantly lower survival rates compared to controls. Histological analysis revealed that *D. vulgaris* exacerbated pathological damage to the liver, kidney, and lung tissues in septic mice. Furthermore, TUNEL staining revealed that *D. vulgaris* increased apoptosis in these tissues. In addition to its effects on these tissues, *D. vulgaris* also exacerbated damage to the intestinal mucosal barrier. The disruption of the intestinal barrier further contributed to the systemic inflammatory response, which is a critical factor in the progression of sepsis (Haak and Wiersinga, 2017). These findings underscore the multi-organ damage caused by *D. vulgaris* and its potential to worsen the overall clinical outcomes of sepsis. This aligns with previous research on the pathogenic role of *Desulfovibrio* species in other inflammatory diseases, particularly inflammatory bowel diseases, where *D. vulgaris* proliferation has been shown to worsen intestinal barrier damage and the secretion of pro-inflammatory cytokines (IL-1β, iNOS, and TNF-*α*), ultimately exacerbating DSS-induced colitis (Huang et al., 2024). Furthermore, previous studies have demonstrated that outer membrane vesicles (OMVs) from *D. fairfieldensis* can impair intestinal epithelial integrity and activate intrinsic inflammatory responses (Nie et al., 2022). *D. desulfuricans* has been reported to aggravate atherosclerosis by increasing intestinal permeability and activating the endothelial TLR4/NF-κB pathway in *Apoe^−/−^* mice (Zhang et al., 2023). Additionally, excessive H₂S production, often associated with gut dysbiosis, has been shown to compromise the intestinal barrier and exacerbate systemic inflammation (Munteanu et al., 2024b).

Beyond local tissue damage, *D. vulgaris* also significantly promotes the systemic inflammatory response in septic mice. We observed a marked elevation in pro-inflammatory cytokines such as TNF-α, IL-1β, and IL-6 in the serum of septic mice, suggesting that *D. vulgaris* may activate the intestinal immune system, thereby enhancing systemic inflammation. Additionally, we found that *D. vulgaris* significantly activated MAPK and NF-κB signaling pathways in peritoneal macrophages of septic mice. Previous study shows that *D. vulgaris* flagellin can activate LRRC19 to trigger NF-κB and MAPK pathways, and subsequently induces the production of pro-inflammatory chemokines and cytokines *in vivo* (Xie et al., 2024). However, an *in vitro* study suggests that *D. vulgaris* may induce the expression of pro-inflammatory cytokines such as TNF-α and iNOS via the PI3K/Akt pathway in a TLR2-dependent manner (Singh et al., 2024), which warrants further investigation.

Another key pathological feature of sepsis is oxidative stress, which exacerbates the pathological progression of the disease (Forman and Zhang, 2021). In our study, *D. vulgaris* significantly increased the levels of ROS in the liver, kidneys, and lungs of septic mice, while significantly reducing antioxidant levels (GSH, CAT, and SOD). We speculate that *D. vulgaris* exacerbates oxidative stress in septic mice by increasing free radical production and inhibiting antioxidant defense mechanisms, which in turn accelerates the progression of multi-organ dysfunction in sepsis. Interestingly, *D. vulgaris* contains genes for encoding superoxide reductase and SOD, which enable it to neutralize superoxide anions and survive in oxygen-rich environments (Fournier et al., 2004; Heidelberg et al., 2004; Zhou et al., 2010). However, in the context of sepsis, *D. vulgaris* may exacerbate oxidative stress. Elevated H₂S levels exhibit cytotoxic effects by inhibiting mitochondrial respiration or increasing ROS generation, thereby amplifying inflammation (Qi et al., 2024). We hypothesize that although *D. vulgaris* can survive in an oxidative environment, certain factors produced by *D. vulgaris*, such as LPS, flagellin, and H_2_S metabolites, could increase the oxidative burden, promoting ROS production and impairing antioxidant defenses, thereby worsening oxidative stress in septic tissues.

Although our study provides valuable insights into the role of *D. vulgaris* in sepsis, several limitations should be acknowledged. We have not fully elucidated the specific pathogenic mechanisms through which *D. vulgaris* exacerbates sepsis, particularly in terms of its effects on immune cell regulation and interactions with other signaling pathways. Additionally, while we focused primarily on *D. vulgaris*, the complexity of gut dysbiosis in sepsis involves a shift in microbial balance, including reductions in commensal bacteria and overgrowth of potential pathogens. *D. vulgaris*-specific bacteriophages could offer a more precise approach, selectively targeting *D. vulgaris* while preserving beneficial gut bacteria (Walker et al., 2006). Dietary interventions may also play a role, as *D. vulgaris* relies on sulfate for metabolism. Reducing sulfur-rich dietary components, such as high animal protein intake, thereby suppressing *D. vulgaris* growth and potentially mitigating organ injury caused by its overgrowth (Zhou L. et al., 2024). Interactions between *D. vulgaris* and other gut microbes may also play a significant role in the progression of sepsis and require further investigation.

## Conclusion

The present study reveals that the increased abundance of *D. vulgaris* in septic mice may exacerbate sepsis through multiple mechanisms, including increased mortality, enhanced inflammation, oxidative stress, and multi-organ dysfunction. *D. vulgaris* likely contributes to systemic inflammation and tissue damage by activating critical signaling pathways such as MAPK and NF-κB. These findings provide a new perspective on the role of the gut microbiome in sepsis and offer potential therapeutic targets for sepsis treatment. Targeting both inflammation and oxidative stress may provide potential therapeutic avenues to mitigate the harmful effects of *D. vulgaris* in sepsis.

## Data Availability

The datasets presented in this study can be found in online repositories. The names of the repository/repositories and accession number(s) can be found in the article/Supplementary material.
