# Supplementary material for: Desulfovibrio vulgaris exacerbates sepsis by inducing inflammation and oxidative stress in multiple organs
Source: Front Microbiol. 2025 Apr 30;16:1574998. doi: 10.3389/fmicb.2025.1574998 (PMC12075292; doi:10.3389/fmicb.2025.1574998)
Supplement: Supplementary file 1 [file Data_Sheet_1.pdf]

Supplementary Material

Desulfovibrio vulgaris Exacerbates Sepsis by Inducing Inflammation and Oxidative Stress in Multiple Organs

Rong Wu[1,#], Zhichao Yu[1,#], Peiheng Guo[1,#], Xiaopeng Xiang[2,#], Yunong Zeng[3], Shanshan Fu[1], Mei Yang[4], Xintao Huang[1], Ze Wang[1], Ali Chen[5], Yuewei Ge[3,*], Xiaoshan Zhao[1,*], Wei Xiao[1,6,*]

[1]School of Traditional Chinese Medicine, Southern Medical University, Guangzhou, 510515, China

[2]Hong Kong University of Science and Technology (Guangzhou), Guangzhou, 511453, China

[3]School of Chinese Materia Medica, Guangdong Pharmaceutical University, Guangzhou, 510006, China

[4]Department of Breast Cancer, Cancer Center, Guangdong Provincial People's Hospital, Southern Medical University, Guangzhou, 510080, China

[5]Center for Drug Research and Development, Guangdong Provincial Key Laboratory for Research and Evaluation of Pharmaceutical Preparations, Guangdong Pharmaceutical University, Guangzhou, 510006, China

[6]Key Laboratory of Glucolipid Metabolic Disorder, Ministry of Education, Guangdong Pharmaceutical University, Guangzhou, 510006, China

[#]These authors contributed equally.

*Corresponding authors: Wei Xiao (xw7688@smu.edu.cn), Xiaoshan Zhao (zhaoxs@smu.edu.cn), and Yuewei Ge (geyuewei@gdpu.edu.cn).

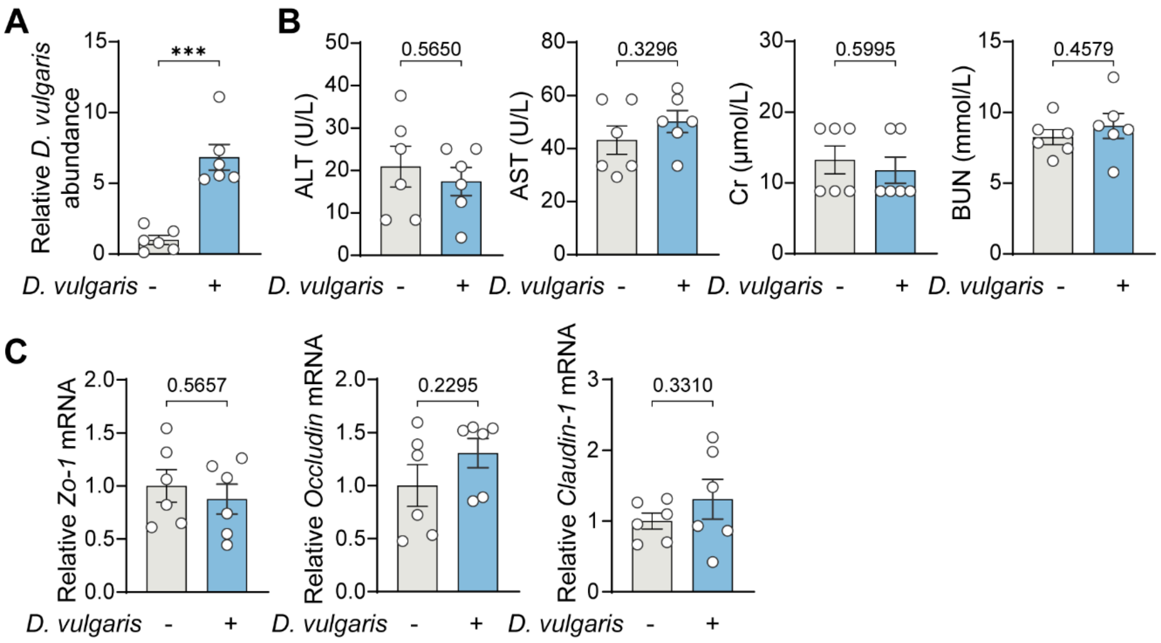

Figure S1. Elevated *D. vulgaris* alone does not cause damage to mice. (A) Abundance of *D. vulgaris* in the feces of mice after transplantation. n=6. (B) Serum levels of ALT, AST, Cr, and BUN in mice with elevated *D. vulgaris* alone. n=6. (C) The relative mRNA levels of *Zo-1*, *Occludin*, and *Claudin-1* in the colon of mice treated with *D. vulgaris* alone. n=6. Data are presented as mean ± SEM. Statistical comparisons were performed using two-tailed unpaired Student's t-tests (A-C). ***p<0.001.

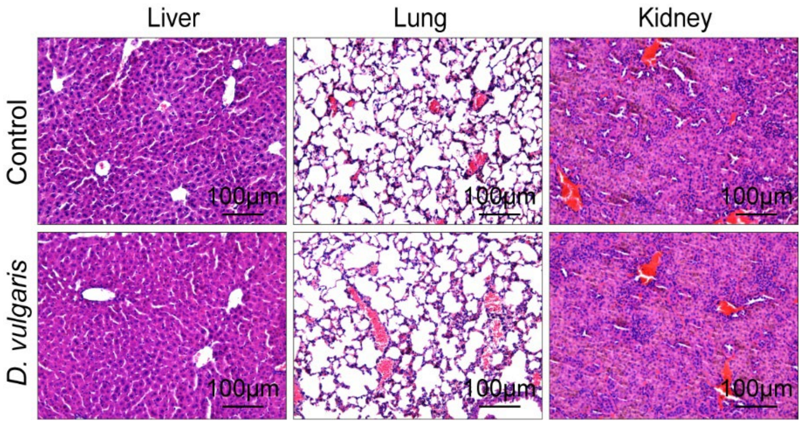

Figure S2. Elevated *D. vulgaris* alone does not cause organ damage in healthy mice.
